# Soft Tissue Infection with *Diaporthe phaseolorum* in Heart Transplant Recipient with End-Stage Renal Failure

**DOI:** 10.3201/eid2509.190768

**Published:** 2019-09

**Authors:** Julia C. Howard, Kevin Chen, Anja Werno, Sarah Metcalf

**Affiliations:** Canterbury Health Laboratories, Christchurch, New Zealand (J.C. Howard, A. Werno);; Christchurch Hospital, Christchurch (K. Chen, S. Metcalf)

**Keywords:** Diaporthe phaseolorum, fungi, fungal infections, immunosuppression, transplant, soft tissue infection, antifungal drugs, heart transplant recipient, end-stage renal failure

## Abstract

*Diaporthe phaseolorum* is a fungal plant parasite that has rarely been described as causing invasive human disease. We report a case of human soft tissue infection with *Diaporthe* *phaseolorum* in a heart transplant patient with end-stage renal failure in New Zealand.

*Diaporthe phaseolorum* is a fungal plant parasite found in soil, salt and fresh water, and sewage (*1*). There are few case reports of human infection with *Diaporthe* species, and most have been described in highly immunosuppressed persons, especially solid organ transplantation recipients (*2**–**6*). One case of *Diaporthe* spp. soft tissue infection was reported in a heart transplant patient in the United States (*5*), but the patient did not have end-stage renal failure (ESRF), making the choice of antifungal therapy less complex. We report a case of human infection with *D. phaseolorum* in a heart transplant patient with end-stage renal failure.

The patient was a 46-year-old man from Samoa, resident in New Zealand, who had had a heart transplant 10 years earlier for dilated cardiomyopathy. We obtained signed consent from the patient for publication of the details of his condition. He had a 1-year history of a slowly enlarging, nontender lump in the pretibial area of his left leg. He noticed the lesion after sustaining a minor abrasion while playing a game of touch rugby. He had not been back to Samoa for 17 years. He was receiving peritoneal dialysis for ESRF secondary to tacrolimus toxicity. At the time of presentation, he was taking mycophenolate (500 mg 2×/d), tacrolimus (5 mg 2×/d; daily trough level 6.6 µg/L), and prednisone (2.5 mg 1×/d).

Magnetic resonance imaging of the leg showed a 37 × 23 × 43 mm complex subcutaneous cystic lesion over the proximal medial tibia with thin septations and no evidence of bony invasion. An aspirate of the lesion was used for microbiologic analysis and culture. Histological examination showed reactive fibroblastic proliferation and numerous fungal hyphae by periodic acid–Schiff staining (Figure).

**Figure:**
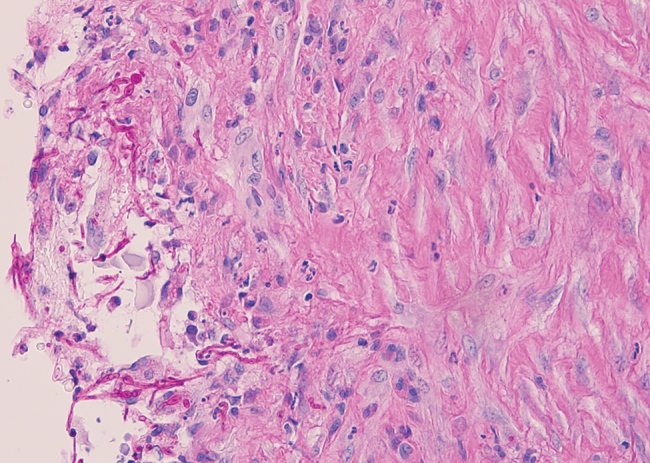
Soft tissue infection with *Diaporthe phaseolorum* in a 46-year-old man from Samoa, resident in New Zealand, who was a heart transplant recipient with end-stage renal failure. Histologic examination of a cystic lesion over the proximal medial tibia showed reactive fibroblastic proliferation and numerous long-branching fungal septate hyphae of uneven widths. Periodic acid–Schiff staining; original magnification ×40. Photograph provided by Frederica Loghides, Department of Anatomic Pathology, Canterbury Health Laboratories.

Microscopy showed numerous fungal hyphae with no identifiable distinctive features by direct microscopic examination. After 3 days of culture, there was growth of small, white, woolly colonies on only chocolate agar and no bacterial growth.

We extracted DNA by using the UCP Pathogen Kit (QIAGEN, https://www.qiagen.com) and sequenced the internal transcribed spacer 2 region. A BLAST query (http://www.ncbi.nih.gov) showed 100% identity and 100% query coverage with *D. phaseolorum* (GenBank accession nos. KX498068.1, KX355829.1, JQ514150.1, and AY577815.1) and *Phomopsis* sp. (GenBank accession nos. GU066693.1 and GQ352481.1, isolates from India and Malaysia).

These findings raised the question of genus assignment. Previously, *Phomopsis* was considered to be the asexual morphotype of *Diaporthe* species. Thus, it is possible that these deposits in GenBank were the same genus and perhaps even the same species. However, without further information about GenBank cultures or morphologic description of our isolate, we can only conclude that our isolate was probably *D. phaseolorum*.

The patient was given voriconazole, and domperidone was replaced with metoclopramide. Severe tremor then developed, so he was given itraconazole before surgical excision of the lesion. Tissue samples grew *D. phaseolorum*. However, drug susceptibility testing could not be performed because of inadequate growth. The patient received itraconazole for 7 months and the infection resolved, with no evidence to date of recurrence.

Infection with *D. phaseolorum* usually occurs after inoculation from direct trauma (*1*). In this patient, the history of a minor leg abrasion during touch rugby was only suggestive of direct inoculation. Previous publications have reported different suspected sources of infection, such as a prick from a plant thorn or spine (*7**,**8*), walking barefoot (*8*), or eye surgery (*9*). Activities that have been implicated in acquisition of infection include gardening (*5**,**7*), farming (*6**,**8*), and hunting (*5*). Another possibility suggested in a case series (*10*) is that the patient might have had a penetrating injury with a wood fragment many years earlier. Marty et al. (*10*) reported 3 cases of cutaneous, invasive fungal disease in which patients had received penetrating soft-tissue injuries with wood fragments months to years (10 months–13 years) before their transplant, suggesting the fungus persisted at the site of injury over a long period.

In the past, diagnosis of this type of unusual fungal infection would be reliant on macroscopic and microscopic morphology and growth characteristics. For this patient, the fungus did not grow on subculture. However, advances in molecular microbiology now enable clinical microbiologists to identify unusual fungal pathogens by sequencing of 18S rDNA or internal transcribed spacer 2 region.

This case was challenging because human infection with this pathogen is rare. Therapeutic options are based on experience detailed in a limited number of case reports. Treatment with itraconazole (*4*,*6**,**8*), posaconazole (*5*), and voriconazole (*3**,**4*) has been successful. However, treatment failure has been reported with voriconazole (despite a low MIC) and terbinafine (*2*). Most case-patients needed surgical resection for infection resolution. Keratitis has been successfully treated with topical amphotericin B and voriconazole (topical and oral) (*7*) and topical natamycin and fluconazole (topical and oral) (*9*).

This case highlights the difficulties faced by clinicians trying to use appropriate directed antifungal therapy when a patient is receiving multiple immunosuppressing drugs. Clinicians and clinical microbiologists should be aware of the possibility of invasive fungal infection with unusual pathogens, even if the patient is seen many years after a transplant.

## References

[1] Sutton DA. Coelomycetous fungi in human disease. A review: Clinical entities, pathogenesis, identification and therapy. Rev Iberoam Micol. 1999;16:171–9. 18473543

[2] Garcia-Reyne A, López-Medrano F, Morales JM, García Esteban C, Martín I, Eraña I, et al. Cutaneous infection by *Phomopsis longicolla* in a renal transplant recipient from Guinea: first report of human infection by this fungus. Transpl Infect Dis. 2011;13:204–7. 10.1111/j.1399-3062.2010.00570.x 21457423

[3] Cariello PF, Wickes BL, Sutton DA, Castlebury LA, Levitz SM, Finberg RW, et al. *Phomopsis bougainvilleicola* prepatellar bursitis in a renal transplant recipient. J Clin Microbiol. 2013;51:692–5. 10.1128/JCM.02674-12 23196359 PMC3553907

[4] Guégan S, Garcia-Hermoso D, Sitbon K, Ahmed S, Moguelet P, Dromer F, et al.; French Mycosis Study Group. Ten-year experience of cutaneous and/or subcutaneous infections due to Coelomycetes in France. Open Forum Infect Dis. 2016;3:ofw106. 10.1093/ofid/ofw106 27419178 PMC4943527

[5] Rakita RM, O’Brien KD, Bourassa L. *Diaporthe* soft tissue infection in a heart transplant patient. Transpl Infect Dis. 2017;19:e12680. 10.1111/tid.12680 28207190

[6] Mattei AS, Severo CB, Guazzelli LS, Oliveira FM, Gené J, Guarro J, et al. Cutaneous infection by *Diaporthe phaseolorum* in Brazil. Med Mycol Case Rep. 2013;2:85–7. 10.1016/j.mmcr.2013.03.001 24432224 PMC3885928

[7] Mandell KJ, Colby KA. Penetrating keratoplasty for invasive fungal keratitis resulting from a thorn injury involving *Phomopsis* species. Cornea. 2009;28:1167–9. 10.1097/ICO.0b013e31819839e6 19770729

[8] Iriart X, Binois R, Fior A, Blanchet D, Berry A, Cassaing S, et al. Eumycetoma caused by Diaporthe phaseolorum (Phomopsis phaseoli): a case report and a mini-review of Diaporthe/Phomopsis spp invasive infections in humans. Clin Microbiol Infect. 2011;17:1492–4. 10.1111/j.1469-0691.2011.03568.x 21781209

[9] Gajjar DU, Pal AK, Parmar TJ, Arora AI, Ganatra DA, Kayastha FB, et al. Fungal scleral keratitis caused by *Phomopsis phoenicicola.* J Clin Microbiol. 2011;49:2365–8. 10.1128/JCM.02449-10 21450952 PMC3122757

[10] Marty FM, Petschnigg EM, Hammond SP, Ready JE, Ho VT, Soiffer RJ, et al. Invasive fungal disease after remote inoculation in transplant recipients. Clin Infect Dis. 2011;52:e7–10. 10.1093/cid/ciq040 21148507

